# Greatest Ascertained Depth of the Ocean

**Published:** 1848-10

**Authors:** 


*Greatest ascertained Depth of the Ocean.*—On the 2d of June, when in latitude 15° 3′ south, and longitude 26° 14′ west, being nearly calm, and the water quite smooth (says Sir James Ross), we tried for, but did not obtain, soundings with 4600 fathoms of line, or 27,600 feet. [Very nearly five miles and a quarter.] This is the greatest depth of the ocean that has yet been satisfactorily obtained. Its determination is a desideratum in terrestrial physics of great interest and importance.—*Lond. Med. Times.*

